# Hot spot detection and spatio-temporal dispersion of dengue fever in Hanoi, Vietnam

**DOI:** 10.3402/gha.v6i0.18632

**Published:** 2013-01-24

**Authors:** Do Thi Thanh Toan, Wenbiao Hu, Pham Quang Thai, Luu Ngoc Hoat, Pamela Wright, Pim Martens

**Affiliations:** 1 Institute of Training for Preventive Medicine and Public Health, Hanoi Medical University, Hanoi, Vietnam; 2 School of Population Health, The University of Queensland, Queensland, Australia; 3 National Institute of Hygiene and Epidemiology of Vietnam, Hanoi, Vietnam; 4 The Medical Committee Netherlands – Vietnam, Amsterdam, the Netherlands; 5 International Centre for Integrated assessment and Sustainable development, Maastricht University, Maastricht, the Netherlands

**Keywords:** dengue fever, hotspots, dynamic dispersion, Hanoi, Vietnam

## Abstract

**Introduction:**

Dengue fever (DF) in Vietnam remains a serious emerging arboviral disease, which generates significant concerns among international health authorities. Incidence rates of DF have increased significantly during the last few years in many provinces and cities, especially Hanoi. The purpose of this study was to detect DF hot spots and identify the disease dynamics dispersion of DF over the period between 2004 and 2009 in Hanoi, Vietnam.

**Methods:**

Daily data on DF cases and population data for each postcode area of Hanoi between January 1998 and December 2009 were obtained from the Hanoi Center for Preventive Health and the General Statistic Office of Vietnam. Moran's I statistic was used to assess the spatial autocorrelation of reported DF. Spatial scan statistics and logistic regression were used to identify space–time clusters and dispersion of DF.

**Results:**

The study revealed a clear trend of geographic expansion of DF transmission in Hanoi through the study periods (OR 1.17, 95% CI 1.02–1.34). The spatial scan statistics showed that 6/14 (42.9%) districts in Hanoi had significant cluster patterns, which lasted 29 days and were limited to a radius of 1,000 m. The study also demonstrated that most DF cases occurred between June and November, during which the rainfall and temperatures are highest.

**Conclusions:**

There is evidence for the existence of statistically significant clusters of DF in Hanoi, and that the geographical distribution of DF has expanded over recent years. This finding provides a foundation for further investigation into the social and environmental factors responsible for changing disease patterns, and provides data to inform program planning for DF control.

Dengue fever (DF), a mosquito transmitted viral infection, is a serious public health concern worldwide, particularly in developing countries. Annually, the number of DF cases has been estimated to range from 50 to 100 million cases worldwide; of which, up to 500,000 cases result in dengue haemorrhagic fever (1, 2). The infection remains a major threat to the community well-being because it is associated with an increased risk of premature mortality and incurs significant health care cost to society (3). In Vietnam, a number of recent outbreaks of DF have also generated significant concerns among international health authorities (4, 5).

As the risk of DF varies with space and time, it is important to have precise knowledge of the regions at risk, the level of risk, risk factors, and the population exposed. In recent years, the development of geographic information systems (GIS) has provided a supportive spatial analytical tool that has enabled epidemiologists to include more simply a spatial component in epidemiologic studies (6). In the field of infectious and vector-borne diseases such as malaria or DF, GIS have been widely used for disease mapping of different pathologies, in analysis of space and space–time distribution of disease data, in identifying risk factors, and in mapping risk areas (5, 7). Moreover, to test whether any clusters can be detected or if the point process is purely randomly distributed, temporal, spatial, and space–time scan statistics (SaTScan) have recently come into common use (8, 9). The advantages of using SaTScan are that it can adjust for confounding variables, it can reduce pre-selection bias as it searches for clusters without specifying their size or location, it gives a single *p*-value as the likelihood-ratio-based test takes account of multiple testing, and finally, it can be applied to a whole region to detect significant clusters in that region (10).

**Summary of Policy Recommendations**

For Hanoi Preventive Medicine Center:

- Hotspots analysis for DF should be widely used in DF surveillance since it can help reallocate the resource to deal with the outbreak more effectively.

For Preventive Medicine System:

- Case-based surveillance system should use GPS data to track the disease outbreak and the effect of intervention.

With increasing concern about the threat posed by DF in Hanoi, a clearer picture of the epidemiology and important risk factors was needed. This study aimed to fill that need. Using spatial scan statistics and GIS, we investigated the spatial distribution of confirmed cases of DF and investigated the areas of high risk within all 14 districts of Hanoi. In addition, we have used GIS and spatial scan statistics to detect the hot spots and identify the disease dynamics dispersion of DF over period between 2004 and 2009 in Hanoi.

## Methods

### Study site

**Fig. 1:**
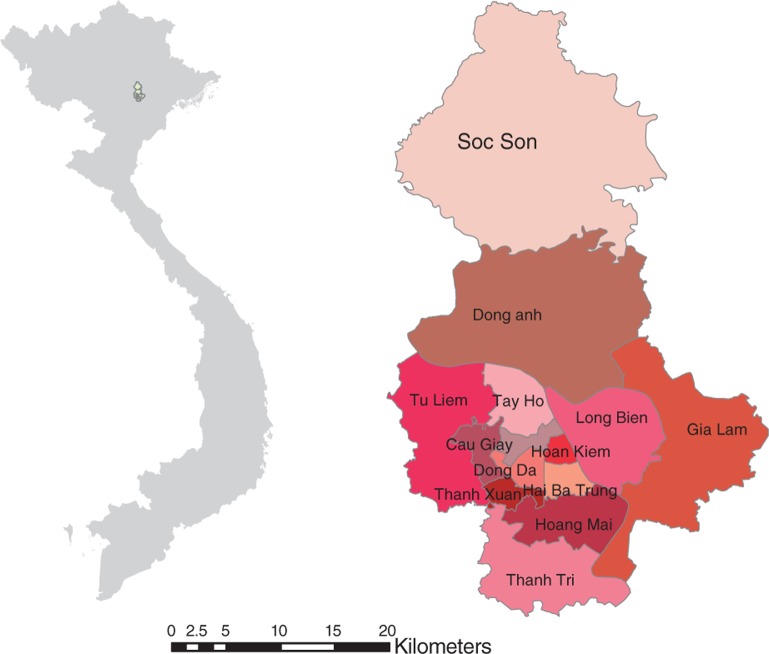
Location of the study area – Hanoi, Vietnam.

Hanoi is located in the north of Vietnam, in the low lying and densely populated Red River delta. Hanoi, before its merging with part of neighbouring provinces in 2008 (known as ‘old Hanoi’), had 14 districts divided into 229 postcode areas. In 2009, the old city had a population of 3.5 million; the population density was quite high, at 1,943 people/km^2^ (11). In this study, the geographic area of ‘old Hanoi’ was selected as a study site because it made it possible to use consistent data for all of the study time periods (2004–2009) (Figure 1).

There is a rapid population growth in Hanoi due to the influx of workers from rural areas. Previous studies in Vietnam showed that people living in poorer areas and old tenement housing, including houses for transient workers, generally tend to have unhygienic conditions with a lack of water supply and absence of window screens, both of which may promote *Aedes* mosquito proliferation and contact. The rapid movement of population between communities may have promoted the largest outbreak of dengue in Hanoi: in 2009, the city recorded almost 8,000 DF patients, representing a 15-fold increase from the previous year.

Hanoi experiences the typical climate of northern Vietnam, where summers are hot and humid, and winters are relatively cool and dry. The summer months from May to September receive the majority of rainfall in the year (1,682 mm rainfall/ year). The winter months from November to March are relatively dry, although spring then often brings light rains. The minimum winter temperature in Hanoi can dip as low as 6–7°C (43–45°F) not including the wind chill, while summer can get as hot as 38–40°C (100–104°F). The period from May to September is suitable for the development of the mosquito vector: high temperatures and high precipitation favour increased rates of mosquito development and a decreased length of reproductive cycle, as well as providing more sites for egg deposition and larva development (12).

With the above characteristics, Hanoi provides very favourable conditions for the existence, circulation and development of infectious diseases such as DF and dengue haemorrhagic fever.

### Data sources

Daily data on DF cases between January 1998 and December 2009 in old Hanoi were obtained from the Hanoi Center for Preventive Health. Data included the onset date and place of onset of the notified cases of DF infection, age, sex, occupation of the patients and laboratory test results. The criteria for notification of DF disease are based on guidelines from the Ministry of Health, 1999, on surveillance, diagnosis, and treatment of dengue, in which individuals suspected to have dengue are those who have acute febrile illness (≥38°C) of 2–7 days duration with two or more of the following non-specific manifestations of DF: headache, retro-orbital pain, myalgia, arthralgia, rash, haemorrhagic manifestations, and leucopenia (11). The total number of cases was 25,483 after excluded cases where the residential address was unspecified (the years 1999–2002 have no addresses). The locations of patient's residence were taken from a Garmin GPS60 (Garmin Corporation, Taipei County, Taiwan) global positioning system while doing a field survey. A total of 51.4% (13,092 cases) of the DF records are available as case event data.

The population data of each postcode area for 2004 and 2009 were obtained directly from the decennial national Population and Housing Census, conducted by the General Statistic Office of Vietnam (GSO; http://www.gso.gov.vn).

### Data analysis

#### Descriptive analysis

Descriptive statistics of numbers of Hanoi areas with notified DF cases in three periods (2004–2005, 2006–2007, and 2008–2009), incidence rate of DF in these periods, or choropleth maps were analysed to describe the dynamics of the disease. The monthly distribution of DF according to seasonality was also performed using boxplots.

#### Spatial autocorrelation analysis

The spatial autocorrelation of the expected incidence rates of DF in three different periods was assessed using Moran's I statistic in the program ArcGIS 9.2. Spatial autocorrelation was considered significant if the *p*<0.05. Moran's I ranges from −1 to 1 and can be interpreted as follows: a value close to 0 indicates spatial randomness, while a positive value indicates positive spatial autocorrelation and a negative value indicates negative spatial autocorrelation.

#### Cluster analysis

Scan statistics were used to detect and evaluate the clusters of cases in either a purely temporal, purely spatial, or space–time setting. This was achieved by gradually scanning a window across time and/or space, noting the number of observed and expected observations inside the window at each location. For each location and size of the scanning window, the alternative hypothesis was that there was an elevated risk within the window compared to outside.

In SaTScan software, the scanning window was an interval (in time), a circle or an ellipse (in space), or a cylinder with a circular or elliptic base (in space–time). Multiple different window sizes were used. The window with the maximum likelihood was the most likely cluster, that is, the cluster least likely to be due to chance. A *p*-value was assigned to this cluster. The standard purely spatial scan statistic imposed a circular window on the map. The window was in turn centred on each of several possible grid points positioned throughout the study region. For each grid point, the radius of the window varied continuously in size from zero to some upper limit specified by the user. In this way, the circular window was flexible both in its location and size, while each circle was a candidate cluster. The space–time scan statistic was defined by a cylindrical window with a circular (or elliptic) geographic base and with its height corresponding to the time. The base was defined exactly as for the purely spatial scan statistic, while the height reflected the time period of potential clusters. The cylindrical window was then moved in space and time, so that for each possible geographical location and size, it also visited each possible time period. In effect, an infinite number of overlapping cylinders of different sizes and shapes were obtained, which jointly cover the entire studied region, where each cylinder reflected a possible cluster. The temporal scan statistic used a window that moved in one dimension, time, defined in the same way as the height of the cylinder used by the space–time scan statistic. This meant that it was flexible in both start and end date. For purely spatial and space–time analyses, SaTScan also identified secondary clusters in the data set in addition to the most likely cluster and lined them up by their likelihood ratio test statistic. For purely temporal analyses, only the most likely cluster was reported. No geographic overlap was used as a default setting, so secondary clusters would not overlap the most significant cluster. In order to scan from small to large clusters, the maximum cluster size was set to 50% of the total population at risk. To ensure sufficient statistical power, the number of Monte Carlo replications was set to 999.

#### Dynamic dispersion of DF

In this study, we try to identify whether changes in DF varied with latitude and longitude of villages centroids in the three periods. Logistic regression models can be constructed with the dichotomous outcome variable defined as whether or not an increase of DF occurred in each village between the three periods. Longitude and latitude of village centroids were entered as explanatory variables. Spatial dispersions can be expressed in terms of odds ratios (OR) for longitude and latitude, with 95% confidence interval (CI).

## Results

### Descriptive analysis

Monthly average numbers of postcode areas with notified dengue cases in Hanoi for three periods 2004–2005, 2006–2007, and 2008–2009 are summarised in Table 1. It was shown that there is a clear trend of geographic expansion of dengue transmission in Hanoi through periods (with mean equal to 41.0, 49.33, and 79.33, respectively).

**Table 1:** Descriptive statistics of monthly numbers of postcode areas with notified dengue cases

Period	Mean	SD	Minimum	Q1^a^	Median	Q3^b^	Maximum
2004–2005	41.00	32.36	5	15.75	36	59	95
2006–2007	49.33	32.47	8	24.25	47	72.25	104
2008–2009	79.33	45.53	17	36.75	74	122.5	146

a Q1, first quartile value.

b Q3, third quartile value.

**Fig. 2:**
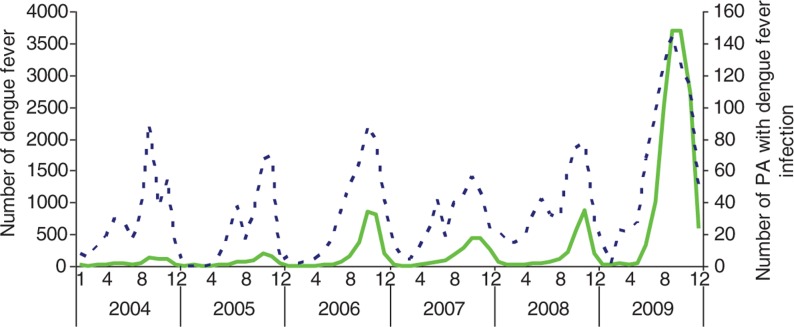
Numbers of dengue cases (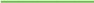) and postcode areas with dengue notifications (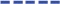) between January 2004 and December 2009 in Hanoi.

Figure 2 presents a striking variation in the monthly numbers of dengue cases and monthly numbers of postcode areas with dengue from 2004 to 2009. A large peak of dengue incidence occurred in October and November 2009 (3,696 cases and 2,698 cases, respectively). Peaks in incident cases coincided with high monthly numbers of postcode areas with DF cases.

**Fig. 3:**
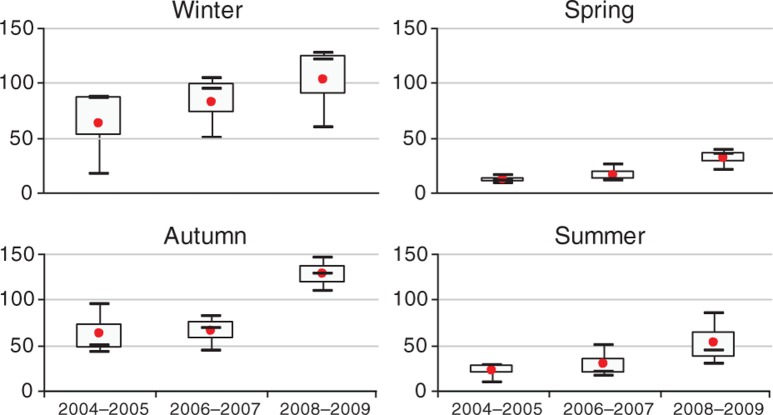
Boxplots of the seasonal distribution of numbers of postcode areas with dengue infection in three periods in Hanoi. The boxplots display the values of the 25th, 50th, and 75th percentiles.

Boxplots of the monthly numbers of postcode areas with dengue are shown in Fig. 3. The results indicated a strongly seasonal pattern (with a peak in autumn and early winter) and suggested that there was an upward trend of dengue incidence from 2004 to 2009.

**Fig. 4:**
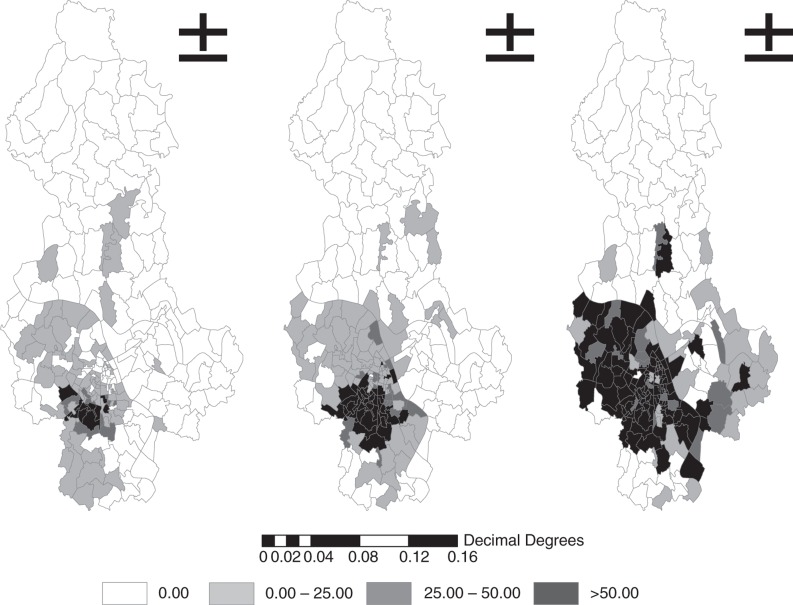
Map showing raw dengue incidence rates in three periods.

Figure 4 shows the geographic distribution of the raw incidence of notified dengue cases in Hanoi in three time periods. There was an expansion of postcode areas with dengue to the west-northern wards in Hanoi between 2004 and 2009. Dengue incidence ranged from 4.55 to 2887.6/100 000 and kept increasing, from 97 to 132 and subsequently 160 postcode areas in the three periods.

### Spatial autocorrelation of DF

A significant positive spatial autocorrelation of dengue incidence for all three periods is presented in Table 2, where Moran's I index was 0.19 (expected Moran's I=−0.011, *p*<0.001) during 2004–2005 (expected Moran's I=−0.007, *p*=0.001), 0.32 during 2006–2007, and 0.22 (expected Moran's I=−0.006, *p*=0.001) during 2008–2009. This means that villages closer together tend to have more similar baseline incidence rates than those further apart.

**Table 2:** Spatial autocorrelation analysis for dengue in Hanoi, 2004–2009

Period	Annual incidence	Moran's I	E(I)	*p*
2004–2005	226.32	0.19	−0.011	0.001
2006–2007	1049.78	0.32	−0.007	0.001
2008–2009	3774.49	0.22	−0.006	0.001

### Purely temporal clustering

The results of the purely temporal clustering analysis in each year also indicate the seasonal tendency of dengue transmission. Table 3 shows the temporal clusters of DF cases in the study area from 2004 to 2009. There were significantly temporal clusters in all years (range of RR from 1.34–14.26), *p*=0.001). The highest RR was found in 2005 (RR: 14.26, *p*=0.001), which suggested there were strongest temporal clusters in 2005. The temporal clusters of DF in Hanoi often covered 6 months (June to December).

**Table 3:** The clusters of dengue cases detected using the purely temporal analysis

Year	Cluster time frame	Total days of cluster	Obs^a^	Exp^b^	Relative risk	LLR^c^	*p*
2004	4/8–1/12	118	216	160.30	1.90	17.68	0.001
2005	5/6–1/12	177	246	167.56	14.26	74.0	0.001
2006	5/6–1/12	177	1,679	1,556.38	2.30	45.39	0.001
2007	5/7–31/12	177	1,051	937.56	1.88	33.36	0.001
2008	4/8–16/12	133	992	886.41	1.60	23.84	0.001
2009	20/6–16/12	179	7,725	7,634.84	1.34	12.64	0.001

a The number of observed cases in a cluster.

b The number of expected cases in a cluster.

c Log likelihood ratio.

### Purely spatial clustering

Analysis of purely spatial clustering of dengue cases from 2004 to 2009, with the maximum spatial cluster size of 50% of the total population, identified the most likely cluster for each of the 6 years. However, only in 2004 and 2005 did the result show a random distribution of dengue in space of Dong Da, Hai Ba Trung, Thanh Xuan and Hoang Mai districts (Table 4). For the years 2006–2009, it was found that the risk is the same inside and outside the cluster since *p>*0.05.

**Table 4:** The clusters of dengue cases detected using the purely spatial analysis

Year		Location	Obs^a^	Exp^b^	Relative risk	LLR^c^	*p*
2004	A	Thanh Tri	175	150.08	1.33	3.56	0.932
2005	A	Hai Ba Trung, Hoang Mai	65	37.10	2.01	10.4	0.002
	B	Thanh Xuan, Dong Da	128	96.19	1.66	8.18	0.017
2006	A	Dong Da, Tay Ho, Long Bien	833	806.05	1.06	0.81	1.000
2007	A	Dong Da, Ba Dinh	658	609.28	1.17	3.89	1.000
2008	A	Tu Liem, Cau Giay	238	215.30	1.13	1.41	1.000
2009	A	Hoan Kiem, Hai Ba Trung	2,346	2323.1	1.02	0.16	1.000

a The number of observed cases in a cluster.

b The number of expected cases in a cluster.

c Log likelihood ratio.

A: Most likely cluster; B: Secondary cluster.

### Space–time clustering

The space–time clustering analysis of the dengue data from 2004 to 2009 was also tested. Figure 4 and Table 5 illustrate the clusters in all districts of Hanoi at a 5% significant level (*p*<0.05) in this period.

**Table 5:** SaTScan statistics for space–time clusters with significantly higher incidence in Hanoi from 2004 to 2009 (most likely cluster)

Location	Radius (km)	Time frame	Relative risk	*p*
Hoang Mai	2.50	4/8–19/7/2004	2.03	0.001
Thanh Xuan	0.68	17/11–31/12/2005	2.66	0.001
Dong Da	0.64	2–16/11/2006	3.51	0.001
Dong Da	0.99	17/11–16/12/2007	3.38	0.001
Hoang Mai	0.29	19/8–2/10/2008	5.58	0.001
Hoan Kiem, Hai Ba Trung	1.56	2–16/11/2009	6.41	0.001

**Fig. 5:**
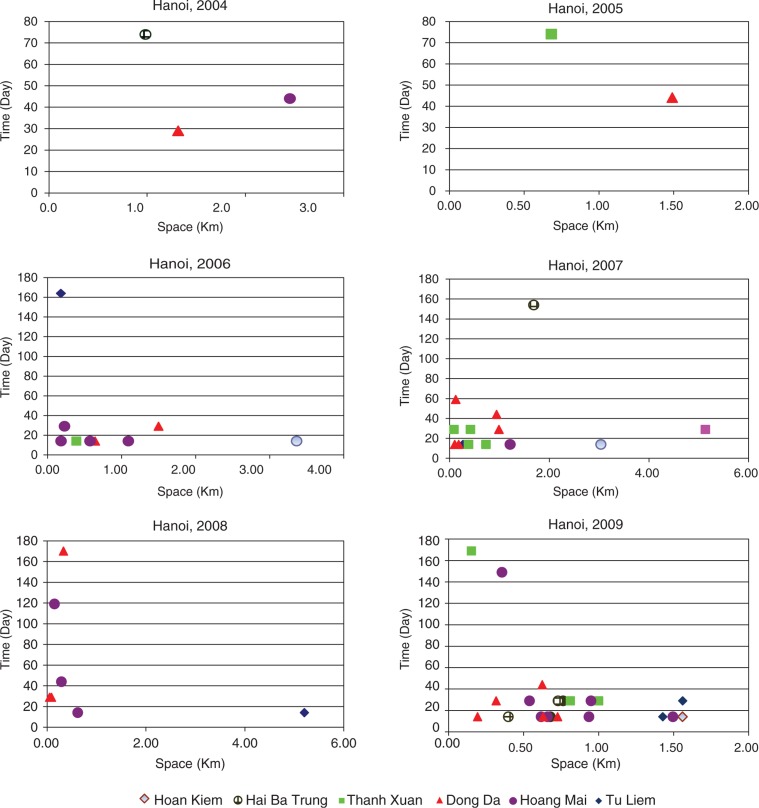
Scatterplot of significant space–time clusters in Hanoi from 2004 to 2009.

The results reveal a high significance of space–time association with DF transmission. It is revealed that six out of the 14 districts of Hanoi had significant cluster patterns, in which Dong Da, Hoang Mai, and Thanh Xuan have the highest number of space–time clusters. The most likely cluster was found to differ during all three year periods. In 2004–2005, only a few clusters were found, distributed over a large distance and time. In 2006–2007, the most likely cluster occurred in Dong Da, with 149 cases and within 14 days in November. In August 2008, the most likely cluster was reported in Hoang Mai and was limited to 250 m (RR=5.58, *p*=0.001). In November 2009, the highest number of dengue cases (553) was again found in Dong Da and Hoan Kiem, within the radius of 1,560 m. The RR within the most likely cluster was 6.41 (*p*=0.001). The secondary clusters reported in Hoang Mai, Tay Ho, and Hai Ba Trung were also limited at 1,000 m and within 29 days (Fig. 5).

## Dynamic dispersion of DF

Logistic regression models were constructed to identify whether changes in DF varied with latitude and longitude of postcode centroids in the three periods (Table 6). The results suggest that changes in DF were significantly associated with latitude (OR 1.17, 95% CI 1.02–1.34) between the periods 2008–2009 and 2004–2005. However, there was no association between DF and longitude in any period.

**Table 6:** Changes of dengue fever in latitude and longitude, Hanoi, 2004–2009

Change in periods	Latitude		Longitude	
	
	OR	95% CI	OR	95% CI
Period 3–Period 1	1.17	1.02–1.34	1.06	0.93–1.34
Period 2–Period 1	0.94	0.85–1.03	0.98	0.87–1.11
Period 3–Period 2	1.09	0.99–1.22	0.98	0.89–1.06

Note: Period 1: 2004–2005; Period 2: 2006–2007; Period 3: 2008–2009.

## Discussion

The results of this study revealed significant spatio-temporal variation in the distribution of DF in Hanoi, Vietnam. Previous studies showed that, in Vietnam, the dengue epidemic often had a cycle of 3–5 years and was expected to reach a 10-year peak (5, 13, 14). These peaks were in 1987, 1998 and 2009, as also recorded in this study, showing an upward trend of dengue cases with the largest outbreaks in 2009. Most of the dengue cases occurred between June and November, when the rainfall and temperature are highest of the year. This time period appeared in our analysis of purely temporal clustering, showing high-risk months for dengue each year between 2004 and 2009. The results are consistent with those of a study conducted in a Central Highlands province of Vietnam, which found that dengue was most prevalent in the wet season (11). As other tropical countries, Vietnam's climate is favourable for the transmission of DF. A warm temperature is crucial to the mosquito's life and gonotrophic cycle, and to virus replication. In addition, stagnant water and higher humidity could augment the epidemic during a rainy season (9, 12, 15–19).

Results from space–time clustering identified the high-risk areas over the larger region and over the years. Using the maximum spatial cluster size of 50% of the total population, and the maximum temporal cluster size of 50% of the total population, we identified six among 14 districts of Hanoi as having significant cluster patterns within a period of 29 days and limited at 1,000 m on average. The areas recording the highest numbers of space–time clusters were Dong Da, Hoang Mai, and Thanh Xuan, with some expansion to the north-western wards of Hanoi, where a higher population is concentrated. Similar results were reported in Malaysia, where geographical weighted regression analysis revealed that the spatial distribution of DF was closely related to population distribution (9). Moreover, the result of their space–time permutation scan statistics showed that most of the clusters were in medium or high population areas. Spatial clustering of disease is almost inevitable, since human populations generally live in spatial clusters rather than random distributions.

Finally, spatial autocorrelation and logistic regression analysis are valuable tools for studying how spatial patterns change over time (20–26). In this study, we found that DF had high spatial autocorrelation in three different time periods. The patterns were closely related to the topography of the environment, in that the villages closer together tended to have more similar baseline incidence rates.

To the best of our knowledge, this is the first study to apply a spatial scan technique, using SaTScan software, to investigate the temporal and geographical clustering of DF disease in Vietnam, in this case in Hanoi. The study provides useful information on the prevailing epidemiological situation of DF in Hanoi city. This new knowledge about the presence of hotspots of DF in the city can help Hanoi Preventive Medicine Center to intensify their remedial measures in the identified areas of high DF prevalence and chalk out future strategies for more effective DF control.

The study does have some potential limitations. The disease case data were not survey-based but used sentinel surveillance data, which only records patients presenting at hospitals. The study analysed statistically significant clusters of DF in Hanoi but did not examine their causes. Future research should focus on the effect of various socio-economic and environmental factors that could affect disease transmission.

## Conclusion and policy implications

The study has shown the presence of long-term hotspots of DF occurrence, which was highest in Dong Da, Hoang Mai, and Thanh Xuan districts of Hanoi. We have also shown the expansion of geographic distribution of DF over recent years. The results demonstrate the necessity to further improve our understanding of the impact of socio-environmental change and ecosystem stress on the transmission of DF. The study has illustrated how, using existing health data, spatial scan statistic and GIS can provide public health officials with necessary information about the prevalence of statistically significant hotspots of DF in the city, thus enabling them to chalk out more effective strategies to contain this scourge. Moreover, hot spot analysis using GIS should be widely used in DF surveillance since it can help reallocate the resource to deal with the outbreak more effectively. This effort will contribute to dengue control strategy.
